# Visual Deprivation Retards the Maturation of Dendritic Fields and Receptive Fields of Mouse Retinal Ganglion Cells

**DOI:** 10.3389/fncel.2021.640421

**Published:** 2021-04-27

**Authors:** Hui Chen, Hong-Ping Xu, Ping Wang, Ning Tian

**Affiliations:** ^1^Department of Ophthalmology and Visual Science, University of Utah School of Medicine, Salt Lake City, UT, United States; ^2^Department of Neurobiology, Yale University School of Medicine, New Haven, CT, United States; ^3^VA Salt Lake City Health Care System, Salt Lake City, UT, United States

**Keywords:** dendritic morphology, development, retinal ganglion cell, activity-dependent plasticity, light deprivation, receptive field (RF)

## Abstract

It was well documented that both the size of the dendritic field and receptive field of retinal ganglion cells (RGCs) are developmentally regulated in the mammalian retina, and visual stimulation is required for the maturation of the dendritic and receptive fields of mouse RGCs. However, it is not clear whether the developmental changes of the RGC receptive field correlate with the dendritic field and whether visual stimulation regulates the maturation of the dendritic field and receptive field of RGCs in a correlated manner. The present work demonstrated that both the dendritic and receptive fields of RGCs continuously develop after eye opening. However, the correlation between the developmental changes in the receptive field size and the dendritic field varies among different RGC types. These results suggest a continuous change of synaptic converging of RGC synaptic inputs in an RGC type-dependent manner. Besides, light deprivation impairs both the development of dendritic and receptive fields.

## Introduction

Retinal ganglion cells (RGCs) receive visual information from bipolar cells (BCs) and amacrine cells (ACs) and convey the visual information to higher centers of the visual system. The function of RGCs is highly disparate, and their functional specificity is determined mainly by their synaptic inputs from presynaptic BCs and ACs. For instance, the dendrites of RGCs are stratified into ON or OFF sublamina of the inner plexiform layer (IPL) of the retina to synapse with ON or OFF BCs, respectively. This dendritic segregation of RGCs in the IPL ensures the ON and OFF RGCs respond to increment or decrement of light stimulation, respectively. Previous studies demonstrated that many morphological and functional properties of RGCs continue to develop after the retina responds to light stimulation. These include the size of dendritic fields (Ren et al., 2010; Qu and Myhr, 2011; Elias et al., 2018), dendritic segregation into ON and OFF layers of the IPL (Tian and Copenhagen, 2003), light-evoked synaptic activity (Tootle, 1993; Wang et al., 2001; He et al., 2011), intrinsic excitability, and spontaneous synaptic inputs (Tian and Copenhagen, 2001; Giovannelli et al., 2008; Qu and Myhr, 2008), and the synaptic connections to BCs (Wang et al., 2001; Tian and Copenhagen, 2003). The development of several of these cellular properties, such as the dendritic segregation of RGCs into ON and OFF layers of the IPL, the spontaneous synaptic inputs of RGCs, and the size of RGC dendritic fields, is regulated by cell activity and visual experience (Tian and Copenhagen, 2001, 2003; Elias et al., 2018).

Although RGC dendrites provide a structure for receiving synaptic inputs from presynaptic cells, whether the development of the dendritic field correlates with the development of the receptive field is inconsistent. Early studies suggested that the receptive field size matched closely to the size of the dendritic field of ON–OFF direction-selective RGCs (DS-RGCs) in rabbits (Yang and Masland, 1992, 1994). However, the size of the receptive field of αRGCs is much bigger than that of the dendritic field in cats (Peichl and Wässle, 1983). In mice, different RGC types show different modes of dendritic growth. Most RGCs exhibit a phase of faster dendritic expansion between postnatal day 8 (P8) and P13, followed by a phase of dendritic retraction between P13 and adulthood (Ren et al., 2010; Elias et al., 2018). However, the morphological αRGCs showed a fast dendritic growing phase but not the dendritic retraction phase, whereas the morphological ON–OFF DS-RGCs expanded at the same pace as the growing retina (Ren et al., 2010). On the contrary, another report shows that the size of the RGC dendritic field increases from P9–14 to P20–24 (Qu and Myhr, 2011). Physiologically, the excitatory centers of RGC receptive fields of cat and rabbit shrink, and the inhibitory surrounds become much more prominent with age (Bowe-Anders et al., 1975; Rusoff and Dubin, 1977). In turtles, the size of the RGC receptive field is small when the retina starts to respond to light and continues to expand until 2–4 weeks post-hatching (Sernagor and Grzywacz, 1995). In mice, the receptive fields of ON and ON–OFF RGCs become smaller after eye opening (Cantrell et al., 2010; Koehler et al., 2011), whereas the receptive field of OFF RGCs decreases during development in one study (Koehler et al., 2011) but not in another study (Cantrell et al., 2010). This study compared the dendritic fields and receptive fields of ON, OFF, and ON–OFF RGCs immediately after eye opening and in young adults.

Previous studies have shown that the functional and morphological maturation of RGCs is regulated by visual experience. Light deprivation retards the developmental increase in RGC spontaneous synaptic inputs, dendritic stratification (Tian and Copenhagen, 2001; Xu and Tian, 2007; Giovannelli et al., 2008), and the number of synapses in the IPL (Sosula and Glow, 1971; Fisher, 1979). Also, light deprivation enlarges the size of the RGC receptive field in turtles (Sernagor and Grzywacz, 1996) and enhances the inhibitory surround of the RGC receptive field in the rat (Di Marco et al., 2009). In mice, dark rearing prevents the developmental consolidation of the dendritic field of J-RGCs (Elias et al., 2018). However, it reduces the receptive fields of ON and OFF RGCs (Akimov and Rentería, 2014). To determine whether light deprivation affects the development of the RGC dendritic and receptive fields in a correlated manner, we examined the dendritic and receptive fields of ON, OFF, and ON–OFF RGCs of mice raised in constant darkness.

## Materials and Methods

### Animals

Transgenic mice expressing yellow fluorescent protein (YFP) in a small fraction of RGCs controlled by Thy1 promoter (Feng et al., 2000) (H line) were used in this study. They were obtained from the Jackson Laboratory (Bar Harbor, ME, United States). In these mice, YFP is expressed by 12 morphological types of RGCs, and these 12 types of RGCs have been well characterized in our previous publication (Xu and Tian, 2007, 2008; Xu et al., 2010). The control animals were housed under 12:12-h cyclic light/dark conditions. Dark-reared animals were housed in a continuously ventilated light-tight box. All the procedures of daily monitoring and routine maintenance of dark-reared mice were conducted under infrared illumination. The handling and maintenance of animals and tissue preparation met the NIH guidelines and were approved by the University of Utah, Committees on Animal Research.

### Preparation of Retinal Whole Mount for Fluorescent Imaging

For the immunostaining, isolated retinas were fixed with 4% paraformaldehyde in PBS for 30 min at room temperature. Fixed retinas were washed for 3 × 10 min in 0.01 M PBS and incubated in 30% sucrose at 4°C overnight. After blocking in 10% normal donkey serum, retinas were incubated in a mixture of a rabbit polyclonal anti-GFP antibody conjugated with Alexa Fluor 488 (1:500) and a sheep polyclonal anti-TH (1:200) for 6 days at 4°C. A secondary antibody (donkey anti-sheep antibody) conjugated with Texas red at 1:50 dilution was used to reveal the anti-TH binding site at 4°C overnight. Then, retinas were flat mounted on Super-Frost Plus slides (Fisher Scientific, Pittsburgh, PA, United States) with Vectashield (Vector Laboratories, Burlingame, CA, United States).

### Confocal Laser Scanning Microscopy

**FIGURE 1:**
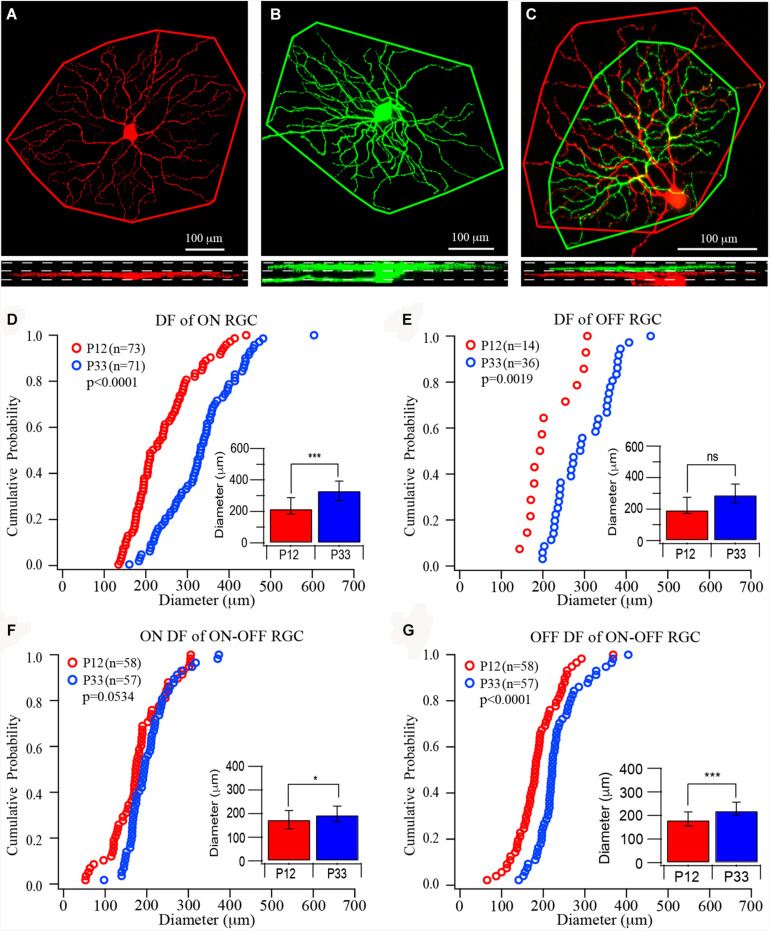
The size of dendritic fields of retinal ganglion cells (RGCs) increases in the postnatal developing mouse retina. **(A–C)** Top: Representative images of the dendritic field of ON, OFF, and ON–OFF RGCs, respectively. The red color shows dendrites ramified in the ON sublamina, and green shows dendrites ramified in the OFF sublamina. Outlines are the measurement of the size of dendritic fields. Bottom: The side view of the three RCCs showing the dendritic distribution in the inner plexiform layer (IPL). (**D–G)** Cumulative distribution curves of the diameter of the dendritic field of ON, OFF, and ON–OFF RGCs of P12 (red) and P33 (blue) mice, respectively. Inset: median diameter of dendritic field. Error bars in the bar graphs indicate interquartile range (IQR). A Mood’s test was used to examine the differences among two or more medians. The *p* values were from K–S tests of cumulative distributions. The “*” sign is used to indicate the *p*-value from Mood tests. *0.05 > *p* > 0.01; ***0.001 > *p*; ns, not significant.

The confocal laser scanning microscopy procedure has been described previously in detail (Xu and Tian, 2007, 2008). Briefly, fluorescent images were collected using a dual-channel Olympus FV5-PSU microscope (Optical Analysis, Nashua, NH, United States) with a PlanApo 60 × oil lens (numerical aperture: 1.4). Image stacks of YFP-expressing RGCs in whole-mount retinas were collected at z-step intervals of 0.5 μm. The dendritic stratification of each RGC was characterized by their ramification depth in the IPL. The IPL was defined as 0–100% from the border of the inner nuclear layer to the border of the ganglion cell layer, determined by the best focus position of the soma of dopaminergic ACs and RGCs, respectively. The dendritic field sizes were measured by the software NeuroExplorer and Neurolucida (MircoBrightField, Williston, VT, United States) based on the stacked image from ON or OFF sublamina, respectively (Figures 1A–C). The dendritic field diameters were calculated from the size of the fields using the equation: $\text{Diameter}=2\times \sqrt{\text{Size}/\text{π}}$.

### Multielectrode Array Recordings and Data Analysis

The procedures of multielectrode Array (MEA) recording have been described previously (Tian and Copenhagen, 2003; Xu and Tian, 2008). Briefly, retinas were isolated from dark-adapted mice under infrared illumination in oxygenated extracellular solution, which contained (in mM) NaCl 124, KCl 2.5, CaCl_2_ 2, MgCl_2_ 2, NaH_2_PO_2_ 1.25, NaHCO_3_ 26, and glucose 22 (pH 7.35 with 95% O_2_ and 5% CO_2_). The isolated retina was mounted on nitrocellulose filter paper, placed in an MEA-60 recording chamber with RGC facing electrodes, and continuously perfused at 34°C. Light-evoked action potential recordings started 90 min after the retinas were positioned in the recording chamber. Action potentials were simultaneously recorded from 60 channels with a MEA having 10-μm diameter electrodes spaced 200 μm apart. The signals were filtered between 100 Hz and 3 kHz.

Visual stimuli were generated on an LCD projector using the software Vision Work on a PC. The images were projected onto the retina through a series of lenses. A small green square (25 μm) was flashing pseudorandomly at different locations (a 60 point × 60 point array) of a test field (1.5 mm × 1.5 mm) at 1 Hz (half second ON and half-second OFF) upon blank background, and the stimulus was repeated three times with a different sequence.

Offline data analysis to isolate the responses from individual neurons was carried out on a personal computer using the software Off-line Sorter (Plexon Inc, Dallas, TX, United States). The timestamps of action potentials recorded by each electrode were stored from the raw data using the Off-line Sorter. The NeuroExplorer (Nex Technologies) was used to plot the perievent histograms of each cell with a 10-ms bin width, and peak time of ON and OFF responses based on the average response to the stimuli on all locations was calculated.

The results were further analyzed with customer-programmed software. First, the response frequency on each location was calculated within 100 ms (peak time ± 50 ms) for ON or OFF responses. In order to decrease the influence of the spontaneous activity, we calculated the spike frequency between 50 ms before the onset or offset of the light and the time of light ON or OFF on each location as spontaneous activity. Then the spontaneous frequency was subtracted from the peak frequency from the same location to have a “calibrated” peak frequency. Finally, the “calibrated” peak frequency at each location was averaged from three trials. If there was only a response to one trial at a location, the average peak frequency was set to zero because the one-time response was most likely a spontaneous, but not a light-evoked, response.

**FIGURE 2:**
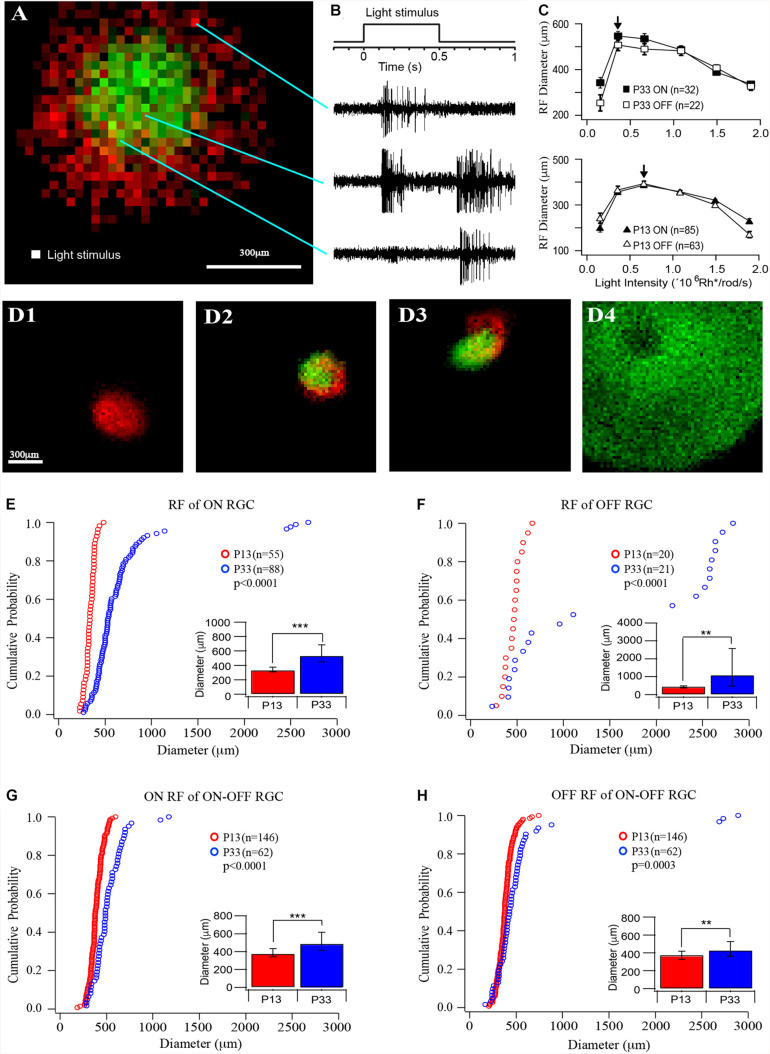
The size of receptive fields of RGCs increases in the postnatal developing mouse retina. **(A)** Representative receptive field map of an ON–OFF RGCs. Red indicates the ON field, and green indicates the OFF field. The pixel size is 25 × 25 μm. **(B)** Light stimulation and representative ON, ON–OFF, and OFF responses evoked by the stimulations at the locations indicated by the lines. **(C)** Plot of the average receptive field diameters versus light intensity for ON (open markers) and OFF (filled markers) fields of P33 (top, squares) and P13 (lower, triangles) mice. The arrows point to the peak diameter locations. **(D)** Four examples of receptive fields of an ON cell (D1), two ON-OFF cells (D2 and D3), and an OFF cell (D4). Red indicates ON receptive fields, and green indicates OFF receptive fields. (**D4)** shows a representative picture of an OFF cell with a regular round but extremely large receptive field and a no-responding center. Because their receptive fields are generally bigger than the entire recording area (1.5 mm × 1.5 mm), we can only record part of the entire receptive field. (**E–H)** Cumulative distribution curves of the diameter of receptive fields of ON, OFF, and ON–OFF RGCs from P12 (red) and P33 (blue) mice, respectively. Inset: median diameter of receptive fields. Error bars in the bar graphs indicate IQR. The *p* values were from K–S tests of cumulative distributions. The “*” sign is used to indicate *p*-value from Mood tests. **0.01 > *p* > 0.001; ***0.001 > *p*; ns, not significant.

The receptive field maps were plotted on a grid (60 × 60 locations, each location is 25 μm × 25 μm) based on the average peak frequency of ON and OFF responses, respectively. Then the receptive field of ON or OFF responses was determined by adding all locations with ON or OFF response. Finally, the diameters of the receptive field were calculated from the size of the fields using the equation: $\text{Diameter}=2\times \sqrt{\text{Size}/\pi }$. For a small group of OFF cells, they have a very regular round but an extremely large receptive field with a no-responding center and a clear margin (Figure 2D4). Their receptive fields are generally bigger than the entire recording area (1.5 mm × 1.5 mm). Therefore, we generally only record part of the entire receptive field and measured the diameter of the receptive field from the no-responding center to the margin.

### Statistical Analysis

The data are presented as cumulative curves and bar graphs in figures. Because some of the data reported in this study are potentially skewed by a small number of very large values, we used median ± interquartile range (IQR) instead of mean ± SE to analyze the differences among the different groups. The IQR describes the middle 50% of the datasets when their value was ordered from lowest to highest. Therefore, the height of the bar indicates the median (the value of the middle point) of the datasets, the upper “error bar” indicates the value of the point at 75% of the datasets, and the lower “error bar” indicates the value of the point at 25% of the datasets. The Kolmogorov–Smirnov (K–S) test was used to determine the significance of the difference in the cumulative distributions, and Mood’s test was used to examine the differences among two or more medians using the statistical add-in, XLSTAT, by Addinsoft to Microsoft Excel (Microsoft). All bar graphs showed in this study are median with IQR.

## Results

### The Size of the Dendritic Field of RGCs Increases After Eye Opening

Previous studies have suggested that the size of the dendritic field of ON and OFF RGCs might be regulated differently during postnatal development (Ren et al., 2010; Qu and Myhr, 2011; Elias et al., 2018). Accordingly, we first quantify the size of mouse RGC dendritic field changes during development after eye opening. To determine whether the development of ON and OFF RGCs are regulated differently, we divided all YFP-expressing RGCs of the transgenic mouse line (Thy1-YFP H line) into three groups, ON, OFF, and ON–OFF RGCs. In the Thy1-YFP (H line) mice, YFP is expressed by 12 morphological types of RGCs, including ON, OFF, and ON–OFF RGCs. These 12 RGC types have been fully characterized in our previous studies (Xu and Tian, 2007, 2008; Xu et al., 2010). We measured the dendritic field diameters of RGCs at the age of P12 and P33, respectively. Because the YFP^+^ RGCs scattered distributed across the entire retina, we sampled all YFP^+^ RGCs with an entire dendritic tree in each retina. The types of ON, OFF, and ON–OFF RGCs are determined based on the depth of their dendritic ramification in the IPL (Figures 1A–C) (Tian and Copenhagen, 2003; Xu and Tian, 2007, 2008; Xu et al., 2010). The sizes of RGC ON and OFF dendritic field were determined based on stacked fluorescent images of Thy1-YFP^+^ RGCs, which were collected with a confocal microscope, from ON or OFF sublamina in the IPL. Figures 1A–C show representative images of an ON, an OFF, and an ON–OFF RGC and the measurement of their dendritic fields.

At the age of P12, the median diameters and the IQRs of the RGC dendritic field are 216.65 and 104.21 μm for ON RGCs (Figure 1D, *n* = 73), 194.5 and 102.06 μm for OFF RGCs (Figure 1E, *n* = 14), 174.37 and 77.12 μm, and 180.56 and 59.18 μm for ON and OFF fields of ON–OFF RGCs (Figures 1F,G, *n* = 58), respectively. The sizes of the RGC dendritic field increase after eye opening. At P33, the median diameters and the IQRs of ON (*n* = 71) and OFF (*n* = 36) RGCs are 331.53 and 123.26 μm, and 289.72 and 122.03 μm, respectively. The ON and OFF RGC dendritic fields increase by 53 and 49%, respectively, from P12 (*p* < 0.0001 for ON RGCs and *p* = 0.115 for OFF RGCs by Mood’s test, respectively, although a K–S test showed a significant difference for the OFF RGCs, *p* = 0.0019, Figures 1D,E). By examining the distribution curves of diameters of dendritic fields of ON and OFF RGCs at P12 and P33, it is evident that the overall distributions of the sizes of dendritic fields of both ON and OFF RGCs shift toward the right side (Figures 1D,E). On the other hand, the median diameters of ON and OFF fields of ON–OFF RGCs (*n* = 58) only increased by 11 and 22% (194.56 and 65.7 μm for ON fields and 220.52 and 55.55 μm for OFF fields, *p* = 0.05 and *p* < 0.0001 for ON and OFF fields, respectively, Figures 1F,G).

### The Size of the Receptive Field of RGCs Increases in a Cell Type-Dependent Manner After Eye Opening

We then measured the sizes of the receptive field of RGCs from mice at the ages of P13 and P33. A MEA system was used to record RGC action potentials evoked by a small square (25 μm × 25 μm) of the light spot, which flash at different locations of a test field (1.5 mm × 1.5 mm) at 1 Hz upon a black background (Figure 2A). The ON and OFF responses were determined based on their response time after lights ON or OFF (Figure 2B). The ON and OFF receptive fields were mapped based on the peak frequency of ON and OFF responses at each location, respectively (Figure 2A). The receptive fields measured in this way only represent the excitatory synaptic inputs (the receptive field center) from ON and/or OFF synaptic pathways, but not inhibitory synaptic inputs from lateral synaptic circuitry (the surround inhibition).

Since the retinas from different ages could have different sensitivity to light, we first examined the light intensity-response profiles of RGCs at P13 and P33 to determine the optimal light intensity with which the RGCs have the maximal size of the receptive field. We stimulated RGCs with six different light intensities (0.154 × 10^6^, 0.359 × 10^6^, 0.664 × 10^6^, 1.09 × 10^6^, 1.49 × 10^6^, and 1.89 × 10^6^ Rh^∗^/rod/s). Figure 2C plots the average receptive field diameters as a function of light intensity for P13 and P33 retinas. With the increase in the stimulus light intensity, the receptive field diameters increase and reach a peak (indicated by an arrow), then decrease for both ON and OFF receptive field. For P33 retinas, the light intensity for maximized receptive field size is 0.359 × 10^6^ Rh^∗^/rod/s, while it is 0.664 × 10^6^ Rh^∗^/rod/s for P13 retinas (Figure 2C). Figure 2D shows four examples of receptive field maps, an ON cell (D1), two ON–OFF cells (D2 and D3), and an OFF cell (D4). Empirically, the size of the receptive field is more consistent in young mice. The adult mice have more RGCs with large receptive fields, especially the OFF RGCs. Interestingly, the ON and OFF receptive fields of many ON–OFF RGCs do not overlap (Figures 2A,D2,D3).

Specifically, the median diameters and the IQR of the receptive field of P13 mice are 337.25 and 66 μm for ON cells (*n* = 55), 471.88 and 121.56 μm for OFF cells (*n* = 20), 379.63 and 92.56 μm, and 378.88 and 90.75 μm for ON and OFF fields of ON–OFF cells (*n* = 146), respectively. The sizes of the receptive field increase after eye opening. At P33, the median receptive field diameters and the IQR of ON and OFF RGCs are 535.63 and 239.94 μm (*n* = 88) and 1,107.3 and 2,095.25 μm (*n* = 21), representing 59 and 135% increase in comparison with P13 mice (*p* < 0.0001 for ON RGCs and *p* = 0.008 for OFF RGCs), respectively. Figures 2E,F show the distribution curves of diameters of the receptive fields of ON and OFF cells at P12 and P33, respectively. The receptive field diameters of P33 cells have a much wider distribution than that of P13 cells (P13: 185–743 μm; P33: 171–2,894 μm). Interestingly, the receptive field diameters of OFF cells were clearly divided into two clusters in the adult mice. The receptive field diameters of one cluster range from 171 to 1,200 μm, and the second cluster ranges from 2,200 to 2,894 μm (Figure 2F). On the other hand, the median diameters and the IQR of ON and OFF fields of ON–OFF RGCs only increased by 29 and 14% (490.25 and 200.38 μm for ON fields and 430.75 and 164.06 μm for OFF fields, *n* = 62, *p* < 0.0001 and *p* = 0.004 for ON and OFF fields, respectively) (Figures 2G,H).

**FIGURE 3:**
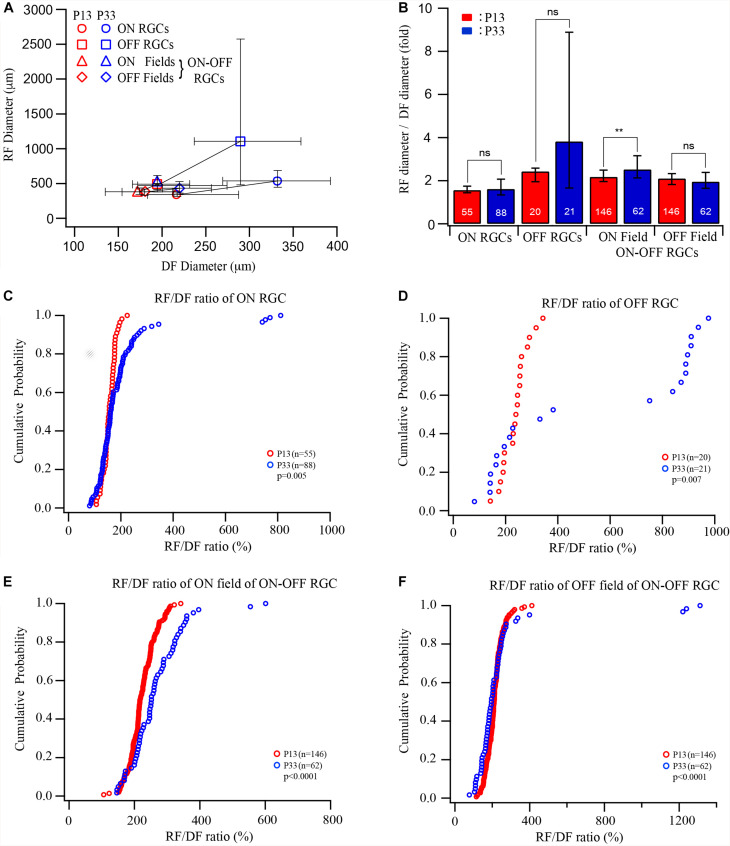
The developmental enlargement of the dendritic and receptive fields develops differently for three RGC groups. **(A)** Median diameters of receptive field plotted as a function of the median diameters of dendritic fields of ON, OFF, and ON–OFF cells of P12/13 and P33 mice. Error bars indicate IQR. **(B)** The ratio of the median diameter of the receptive/dendritic field of ON, OFF, and ON–OFF cells of P12/13 and P33 mice. Because we did not simultaneously measure the diameters of the receptive field and dendritic field of the same RGCs in this study, we divided the receptive field diameter of each cell by the median of the dendritic field of the RGC group at the same age to calculate the ratio of receptive/dendritic field. The numbers in each bar indicate the number of cells. **0.01 > *p* > 0.001; ns, not significant. (**C–F)** Cumulative distribution curves of the receptive/dendritic field ratios of ON, OFF, and ON–OFF cells of P13 and P33 mice. The *p* values were from K–S tests.

The results showed above demonstrated that the sizes of both the dendritic and receptive fields increase after eye opening. However, the developmental increase in the size of the receptive field and the dendritic field varies among different RGC types. These results suggest that the extent of synaptic convergence of RGCs might be regulated in an RGC type-dependent manner during postnatal development. Figure 3A plots the median diameters of the receptive fields of ON, OFF, and ON–OFF cells as a function of the median diameters of dendritic fields of P12–13 and P33 cells. To compare the receptive/dendritic field ratio of RGCs, we divided the receptive field diameter of each cell by the median of the dendritic field of the RGC group at the same age to calculate the ratio of the receptive/dendritic field of each cell. This is because we did not simultaneously measure the diameters of the receptive field and dendritic field of the same RGCs in this study. We then calculated the median and IQR of the receptive/dendritic field ratio for each group. Figure 3B plots the median receptive/dendritic field ratio and IQR of ON, OFF, and ON–OFF cells of P12–13 and P33 mice. The median diameters of the dendritic field of ON and OFF RGCs increased by 0.53- and 0.49-fold from P12 to P33. On the other hand, the median diameter of the receptive field of ON and OFF RGCs increased by 0.59- and 1.35-fold from P13 to P33. These resulted in a minimum change in the receptive/dendritic field ratio of ON RGCs (from 1.55- to 1.61-fold) but a 1.4-fold increase in the receptive/dendritic field ratio of OFF RGCs (from 2.43- to 3.82-fold), respectively (Figure 3B and Table 1). However, the diameter of the ON and OFF dendritic fields of ON-OFF RGCs only increased by 0.34-fold and decreased by 0.15-fold from P12 to P33, respectively (Table 1). Similarly, the median diameter of the ON and OFF receptive field of ON–OFF RGCs only increased by 0.29- and 0.14-fold from P12 to P33 (Table 1). Therefore, the receptive/dendritic field ratio of the ON field of ON–OFF RGCs increased by 0.34-fold (from 2.18- to 2.52-fold), while the OFF field of ON–OFF RGCs decreased by 0.15-fold (from 2.1- to 1.95-fold) from P12 to P33 (Figure 3B and Table 1). We tested the differences of the medians of the receptive/dendritic field ratio between P12–13 and P33 RGCs using Mood’s test. Only the difference of the median diameter of the ON receptive field of ON–OFF RGCs between P12–13 and P33 RGCs is statistically significant (*p* = 0.004, Figure 3B). However, K–S tests showed significant differences in the receptive/dendritic field ratio between P12–13 and P33 RGCs for all three RGC groups (Figures 3C–F).

**TABLE 1:** Size of dendritic field and receptive field of retinal ganglion cells (RGCs) at P12/13 and P33.

Cell type		P12–13 (median and IQR)	P33 (median and IQR)	Ratio (P33/P12)
ON cells	DF	216.7 and 104.2 μm	331.5 and 123.3 μm	1.53
	RF	337.3 and 66 μm	535.6 and 239.9 μm	1.59
	RF/DF ratio	1.55	1.61	
OFF cells	DF	194.5 and 102.1 μm	289.7 and 122 μm	1.49
	RF	471.9 and 121.6 μm	1,107 and 2,095.3 μm	2.35
	RF/DF ratio	2.43	3.82	
ON of ON–OFF cells	DF	174.4 and 77.1 μm	194.6 and 65.7 μm	1.12
	RF	379.6 and 92.6 μm	490.3 and 200.4 μm	1.29
	RF/DF ratio	2.18	2.52	
OFF of ON-OFF cells	DF	180.6 and 59.2 μm	220.5 and 55.6 μm	1.22
	RF	378.9 and 90.8 μm	430.8 and 164.1 μm	1.14
	RF/DF ratio	2.1	1.95	

In conclusion, the ratio of the receptive/dendritic field of all three RGC groups has significantly altered the distribution pattern, while the median analysis only shows a significant difference in the ON field of ON–OFF cells. Therefore, these results suggest that the synaptic converging seems to develop differently for different RGC types or synaptic pathways after eye opening.

### Visual Deprivation Retards the Maturation of Dendritic and Receptive Fields of RGCs

Previous studies demonstrated that light deprivation retarded the developmental refinement of RGC synaptic activity, the RGC dendritic distribution in the IPL, the number of synapses in the IPL, and the development of RGC dendritic and receptive fields (Sosula and Glow, 1971; Fisher, 1979; Sernagor and Grzywacz, 1996; Tian and Copenhagen, 2001, 2003; Xu and Tian, 2007; Giovannelli et al., 2008; Di Marco et al., 2009; He et al., 2011; Akimov and Rentería, 2014; Elias et al., 2018). However, the effects of light deprivation on the size of the RGC receptive field seem to be contradictory. It was reported that light deprivation enlarged the size of the RGC receptive field in turtle when the receptive field was measured using flashing spots (Sernagor and Grzywacz, 1996). In mice, light deprivation causes ON and OFF RGCs to have a smaller receptive field when the receptive field was measured using white noise checkerboard stimulus (Akimov and Rentería, 2014). Because the white noise checkerboard stimulates the whole retina, it might affect the RGC response differently compared with the flashing spot. Therefore, it is still an open question whether the difference in the effects of visual deprivation on the development of RGC receptive fields is due to the difference in species or the stimulus. Besides, it needs to be further illustrated whether the developmental change in the receptive fields of RGCs is consistent with the developmental change in the dendritic fields. Accordingly, we examined whether light deprivation alters the development of the receptive field and dendritic field of RGCs proportionally.

**FIGURE 4:**
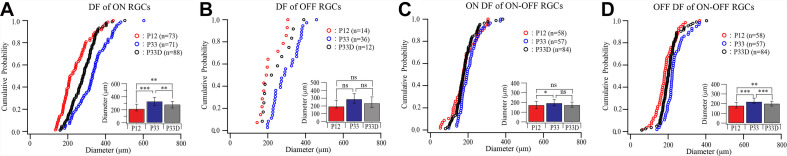
Visual deprivation retards the developmental enlargement of dendritic fields of RGCs. **(A–D)** Cumulative distribution curves and median diameters of dendritic fields of P12 and P33 mice raised in cyclic light–dark condition and P33 mice raised in constant darkness (P33D) from birth for ON, OFF, and ON–OFF RGCs, respectively. Inset: median diameters of dendritic fields. Error bars in the bar graphs indicate IQR. *0.05 > *p* > 0.01; **0.01 > *p* > 0.001; ***0.001 > p; ns, not significant.

We first raised the Thy1-YFP mice in the darkness from birth to P33 and examined the dendritic field of YFP-expressing RGCs. Figure 4 shows that the sizes of dendritic fields of all three groups of RGCs are reduced in dark reared mice. The median dendritic field diameters of ON and OFF RGCs of dark reared mice are 85% (331.53 and 123.26 μm for median and IQR of normally reared P33 ON RGCs, *n* = 71; 282.36 and 100.58 μm for median and IQR of dark reared P33 ON RGCs, *n* = 88, *p* = 0.001, Mood test, Figure 4A) and 81% (289.72 and 122.03 μm for median and IQR of normally reared P33 OFF RGCs, *n* = 36; 235 and 134.05 μm for median and IQR of dark reared P33 OFF RGCs, *n* = 12, *p* = 0.739, Mood test, Figure 4B) of that of age-matched controls, respectively. The K–S tests show a significant difference in the distributions of dendritic field size between dark reared P33 ON RGCs and age-matched controls (*p* = 0.001) but an insignificant difference between dark reared P33 OFF RGCs and age-matched controls (*p* = 0.088) (Figures 4A,B). The median dendritic field diameters of ON and OFF fields of ON–OFF RGCs of dark reared mice are 90% (194.56 and 65.7 μm for median and IQR of ON field of ON–OFF RGCs in normally reared P33 mice, *n* = 57; 175.91 and 51.79 μm for median and IQR of ON field of ON–OFF RGCs in dark reared P33 mice, *n* = 84, *p* = 0.074, Mood test, Figure 4C) and 91% (220.52 and 55.55 μm for median and IQR of OFF field of ON–OFF RGCs in normally reared P33 mice, *n* = 57; 201.12 and 48.84 μm for median and IQR of OFF field of ON-OFF RGCs in dark reared P33 mice, *n* = 84, *p* < 0.0001, Mood test, Figure 4D) of that of age-matched controls, respectively. Consistently, the K–S tests show an insignificant difference in the distributions of dendritic field size between ON field of ON–OFF RGCs in dark reared P33 mice and age-matched controls (*p* = 0.056) but a significant difference between OFF field of ON–OFF RGCs in dark reared P33 mice and age-matched controls (*p* < 0.0001) (Figures 4C,D). Therefore, light deprivation seems to have a more significant effect on ON cells’ dendritic field and the OFF field of ON–OFF cells.

**FIGURE 5:**
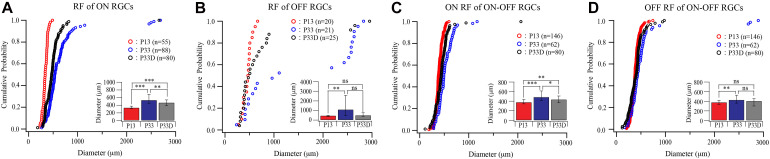
Visual deprivation retards the developmental enlargement of receptive fields of RGCs. **(A–D)** Cumulative distribution curves and median diameters of receptive fields of P13 and P33 mice raised in cyclic light–dark condition and P33 mice raised in constant darkness (P33D) from birth for ON, OFF, and ON–OFF RGCs, respectively. Inset: median diameters of the receptive field. Error bars in the bar graphs indicate IQR. *0.05 > *p* > 0.01; ** 0.01 > *p* > 0.001; ***0.001 > *p*; ns, not significant.

Similarly, light deprivation also reduced the developmental expansion in the size of receptive fields in an RGC type-specific manner (Figure 5). Specifically, the median receptive field diameters of the receptive field of ON and OFF RGCs of dark reared mice are 88% (535.63 and 239.94 μm for median and IQR of normally reared P33 ON RGCs, *n* = 88; 470.5 and 152.06 μm for median and IQR of dark reared P33 ON RGCs, *n* = 80, *p* = 0.002, Mood test, Figure 5A) and 46% (1,107 and 2,095.25 μm for median and IQR of normally reared P33 OFF RGCs, *n* = 21; 508 and 365.5 μm for median and IQR of dark reared P33 OFF RGCs, *n* = 25, *p* = 0.076, Mood test, Figure 5B) of that of age-matched controls, respectively. However, the differences in the distributions of receptive field diameters of both dark reared ON and OFF RGCs are significantly different from that of age-matched controls (*p* = 0.004 and *p* = 0.019 for ON and OFF RGCs, K–S test, Figures 5A,B).

The median receptive field diameters of ON and OFF fields of ON–OFF RGCs of dark reared mice are 89% (490.25 and 200.38 μm for median and IQR of ON field of ON–OFF RGCs in normally reared P33 mice, *n* = 62; 438.13 and 142.25 μm for median and IQR of ON field of ON–OFF RGCs in dark reared P33 mice, *n* = 80, *p* = 0.011, Mood test, Figure 5C) and 94% (430.75 and 164.06 μm for median and IQR of OFF field of ON–OFF RGCs in normally reared P33 mice, *n* = 62; 406.88 and 151.38 μm for median and IQR of OFF field of ON–OFF RGCs in dark reared P33 mice, *n* = 80, *p* = 0.398, Mood test, Figure 5D) of age-matched controls, respectively. Consistently, the K–S tests show a significant difference in the distributions of receptive field size between ON field of ON–OFF RGCs in dark reared P33 mice and age-matched controls (*p* = 0.006) but an insignificant difference between OFF field of ON–OFF RGCs in dark reared P33 mice and age-matched controls (*p* = 0.059) (Figures 5C,D). These results demonstrate that both the developmental expansion of the RGC dendritic field and receptive field are regulated by visual experience.

## Discussion

In this study, we investigated the relationship of the size of the dendritic field and receptive field of RGCs in developing mouse retina and the effect of visual stimulation on the development of RGC dendritic field and receptive field. We show that both the dendritic field and receptive field of RGCs are relatively small at the time of eye opening, but the size of the receptive field is larger than the dendritic field. After eye opening, the RGCs extend the sizes of both the dendritic and receptive fields in a cell type-dependent manner. We also show that light deprivation retards the development of both the dendritic and receptive fields of RGCs.

Retinal ganglion cells respond to light in a restricted region of the retina, which is defined as the receptive field of the cell. Typically, the receptive field of RGCs has a center and a concentric, antagonistic surround and is subdivided into two types: ON-center and OFF-center receptive fields. For an ON-center cell, a spot of light covers the center of the field causing the cell fire spikes. The frequency of the responses increases with the enlargement of the spot size until it reaches the center size of the receptive field. When the spot size is larger than the receptive field center size, the cell responses will decrease. This is because the stimulation of the surrounding portion of the receptive field could inhibit the center of the field. The receptive field of the OFF cell is the mirror image of that of the ON cell (Sagdullaev and Mccall, 2005). This receptive field organization of RGCs results from inputs that arise through both the vertical and lateral pathways in the retinal circuit. Specifically, the antagonistic surround arises in the outer retina at the bipolar cell level via inhibitory inputs from horizontal cells (Mangel, 1991). Surround inhibition is then tuned by amacrine cell-mediated lateral inhibition in the inner retina (Cook and McReynolds, 1998; Taylor, 1999; Flores-Herr et al., 2001; Sinclair, 2004). Besides, direct inhibition via amacrine cell input also has been shown in RGCs (Belgum et al., 1987; Merwine et al., 1995; Flores-Herr et al., 2001; Zaghloul et al., 2003).

Retinal ganglion cell dendrites provide a structure for receiving synaptic inputs from presynaptic cells. However, reports on the correlation between the size of the RGC dendritic field and the receptive field seem to be inconsistent. Some studies suggested that the size of the receptive field matched closely to the size of the dendritic arbor (Yang and Masland, 1992; Kim et al., 2008; Huberman et al., 2009). For instance, the receptive field of ON–OFF DS-RGCs in rabbits is only 6% larger than their dendritic field when measured using moving bars (Yang and Masland, 1994). Also, when comparing the receptive field with the dendritic arbor of individual RGCs, the fine structure of the receptive field of RGCs is defined by the interactions between an RGC dendritic tree and the local mosaic of bipolar cell axons (Brown et al., 2000). This is supported by other earlier studies (Cohen and Sterling, 1991; Kier et al., 1995) and a computational model (Freed et al., 1992). On the other hand, some reports suggested that the size of the receptive field does not match the size of the dendritic arbor. For instance, the receptive field of αRGCs in cats is 40% larger than their dendritic field when measured using flicking spot stimulation (Peichl and Wässle, 1983). A more recent study of mice showed that RGC dendritic field is generally 16% larger than their receptive field when the retina was stimulated using a white noise checkerboard, and the size of the RGC receptive field was determined by STC-NC analysis (Cantrell et al., 2010).

In our results, the level of correlation between dendritic field and receptive field varies significantly among cell types. In adult mice, the ON cells and the ON and OFF fields of ON–OFF cells have a closer correlation between the dendritic field and receptive field (the dendritic/receptive field ratio varies from 1.6 to 2.5). In contrast, the OFF cells have a much bigger dendritic/receptive field ratio (3.8). Clearly, the sizes of the receptive field of OFF cells are divided into two clusters in the adult mice; a group of OFF cells has an unusually large receptive field. This unusual large receptive field does not correlate to the size of the dendritic field of OFF cells. The unusual large field OFF RGCs has been identified in the rabbit retina using spot stimulation (Barlow et al., 1964). These large-field OFF RGCs in the rabbit retina take about 11% of the total RGCs examined, and the diameter of the receptive field of these large-field OFF RGCs is 2.8 times bigger than the receptive field of ON–OFF DS-RGCs. In our study, these large-field OFF RGCs take about 50% of OFF RGCs examined and 6% of total RGCs examined. The median diameter of the receptive field of these large-field OFF RGCs (Q3, 2,575 μm) is 5.4 times bigger than the median diameter of the receptive field of the OFF cells with the smaller receptive field (Q1, 480 μm). Because no RGC with such a big dendritic field has been identified, how these large-field OFF RGCs receive inputs from such a large area is not clear. One possibility is that the electrical coupling between photoreceptors and horizontal cells conveys the light signals in a larger area into a single RGC (Barlow et al., 1964). Also, because we did not match the dendritic field with the receptive field of each cell recorded, we could not rule out the possibility that some ACs with large receptive fields might be recorded in this study.

We compared the receptive field measured using spot stimulation in our study with two recent studies using white noise checkerboard stimulation. One study found that the average diameters of the receptive field for ON and OFF RGCs were 154 ± 2.0 and 154 ± 1.8 μm in P30–39 mice, respectively (Koehler et al., 2011). Another study showed that the average diameters of the receptive field for ON and OFF RGCs corresponded to 140 and 134 μm in P18 mice (Cantrell et al., 2010). The results of these two studies are relatively consistent. In our results, the median diameters of the receptive field of ON and OFF RGCs were 535.63 and 1,107 μm in P33 mice, which are much bigger than those reported by those two studies. Currently, it is not clear what causes this discrepancy. However, there are at least two possibilities that could be accounted for this discrepancy. First, the white noise checkerboard stimulates both the excitatory center and the inhibitory surrounding of the receptive field. Activation of the inhibitory surrounding could reduce the size of the excitatory center of the receptive field. In our study, the single-spot stimulation will not simultaneously stimulate the excitatory center and the inhibitory surrounding of a receptive field and, therefore, will not inhibit the excitatory center of the receptive field when the center was stimulated. Second, with the constant stimulation of the whole retina, the white noise checkerboard could bleach the rods and, therefore, reduce the sensitivity of RGCs and have a smaller excitatory receptive field. In our study, each location of the retina was only stimulated three times (0.5 s at each time) during the entire recording. Therefore, there was minimum bleaching of the photoreceptors.

It is well known that RGC dendrites undergo significant refinement during postnatal development in mice. This includes both the refinement of dendritic ramification into the ON and OFF layers of the IPL and the refinement of the size of the dendritic field. We have previously reported that the developmental refinement of RGC dendritic ramification in the IPL significantly altered the populations of morphologically identified OFF and ON–OFF cells. For instance, the population of RGCs ramified in both the ON and OFF layers of IPL decreased from 41–53% at P12 to 29–32% at P28–30, which is accompanied by an increase in OFF RGCs from 9 to 21% (Tian and Copenhagen, 2003; Xu and Tian, 2007). Similarly, it was shown that the population of morphologically identified ON–OFF RGCs decreased from 66% at P10 to 31% at P30 (Landi et al., 2007) and from 50% at P13 to 35% at P28 (Liu et al., 2007). Consistent with the morphological changes, we previously showed that the population of ON–OFF cells physiologically identified by full-field light stimulation decreased from 41% at P13–15 to 22% at P27–30 (Tian and Copenhagen, 2003). Similarly, the population of ON–OFF cells identified by white noise checkerboard stimulation decreased from 35% at P18 to 24% at P25 (Cantrell et al., 2010). In the current study, we show that the ON–OFF cells identified by spot stimulation decrease from 66% at P13 to 36% at P33. Also, about 50–60% RGCs are ON RGCs, and 5–15% are OFF cells in adult mice in previous reports and our current study (Tian and Copenhagen, 2003; Liu et al., 2007; Cantrell et al., 2010). Therefore, the developmental refinement of RGC dendritic ramification in the IPL is consistent with the synaptic inputs from ON and OFF BCs.

However, the developmental refinement of the size of the RGC dendritic field seems to vary dramatically among different RGC types, and the correlation between the developmental refinement of the RGC dendritic field and receptive field from different studies seems to be inconsistent. For instance, some RGC types exhibit a phase of fast dendritic expansion between postnatal day 8 (P8) and P13, followed by a phase of dendritic retraction between P13 and adulthood (Ren et al., 2010; Elias et al., 2018). Other RGC types, such as αRGCs, showed a fast phase of dendritic growth but not the phase of dendritic retraction. On the other hand, the morphological ON–OFF DS-RGCs continuously expand the size of their dendritic fields at the same pace as the growth of the retina (Ren et al., 2010). In other reports, the size of the RGC dendritic field increases from P9–14 to P20–24 without retraction (Qu and Myhr, 2011), and the dendritic field size of ON RGCs increases almost 47% in 2 weeks after eye opening (Cantrell et al., 2010).

Although the receptive field of RGCs is developmentally regulated as that of the RGC dendritic field, whether the size of the receptive field change with age as that of the dendritic field is debatable. It was reported that the excitatory centers of RGC receptive fields in cat and rabbit shrink, and the inhibitory surrounds become much more prominent with age (Bowe-Anders et al., 1975; Rusoff and Dubin, 1977). In turtle, the size of the RGC receptive field is small at the time the retina starts to respond to light and continue to expand until 2–4 weeks post-hatching (Sernagor and Grzywacz, 1995). In mice, the receptive field centers of ON and ON–OFF RGCs become smaller after eye opening, although the dendritic field size of ON RGCs increases by 47% during the same period (Cantrell et al., 2010; Koehler et al., 2011). However, the receptive field of the OFF RGCs is decreased in one study (Koehler et al., 2011) but not in another (Cantrell et al., 2010).

In this study, we show that both the dendritic field and receptive field of RGCs are relatively small before eye opening, but the size of the receptive field is a factor of 1.5–2.4 larger than that of the dendritic field at P12–13. After eye opening, RGCs extend the size of the dendritic field by 12–53% from P12 to P33, while the sizes of receptive fields are increased by 14–134%. Therefore, the proportions of the increase in the dendritic field and receptive field of ON, OFF, and ON–OFF cells are significantly different. More specifically, the diameters of dendritic fields of ON and OFF RGCs increase by 53 and 49%, while the diameters of receptive fields of ON and OFF RGCs increase by 59 and 135%. However, the ON and OFF dendritic fields of ON–OFF RGCs only increase by 12 and 22%, and the ON and OFF receptive fields of ON–OFF RGCs only increase by 29 and 14%. Therefore, our results indicate a cell type-dependent continuous increase in synaptic converging of RGCs, especially a group of large-field OFF cells. Interestingly, the large-field OFF cells were not detected in P13 mice, suggesting that the synaptic converging of the large-field OFF cells develops after eye opening. Nonetheless, it should be noticed that some of the inconsistencies observed between developmental changes in RGC dendritic morphology and physiology could be merely attributable to a different sampling of RGC subtypes in morphological and physiological studies. This different sampling might be due to that only 12 RGC types express YFP in the Thy1-YFP (H line) mice (Xu and Tian, 2007, 2008; Xu et al., 2010), and these 12 RGC types might not be proportionally labeled with YFP, while the MEA experiments record indiscriminately from all RGC types. However, the developmental changes in RGC dendritic morphology and physiology between different ages and rearing conditions are less likely to be affected by this sampling difference.

It is well demonstrated that visual experience is required for the developmental regulation of RGC dendritic ramification in the IPL and synaptic connection with ON and OFF BCs. We previously showed that light deprivation retarded the RGC dendritic segregation into ON and OFF layers of the IPL (Tian and Copenhagen, 2003; Xu and Tian, 2007). Also, long-term blockage of ON bipolar cell activity by injection of APB into cat’s eyes induced similar effects as light deprivation (Deplano et al., 2004). It is less consistent about the role of visual experience on the development of the size of the RGC dendritic field and receptive field. Light deprivation enlarges the size of the RGC receptive field to more than twice of normally reared turtles (Sernagor and Grzywacz, 1996) and enhances the inhibitory surrounding of the RGC receptive field in the rat (Di Marco et al., 2009). In mice, dark rearing prevents the developmental consolidation of the dendritic field of the J-RGCs between P13–30 (Elias et al., 2018). However, it reduces the ON and OFF receptive field of RGCs (Akimov and Rentería, 2014). Currently, little is known whether visual experience regulates the development of RGC dendritic and receptive fields in a correlated manner.

Our results show that light deprivation retards the developmental increase in RGC dendritic and receptive fields of mice after eye opening. Compared with age-matched controls, the median dendritic fields of ON and OFF RGCs are reduced by 15 and 19% in dark reared mice, while the receptive fields of ON and OFF RGCs are reduced by 12 and 54% in the dark reared mice. For the ON–OFF RGCs, the dendritic fields of ON and OFF fields are reduced by 10 and 9% in dark reared mice, and the receptive fields of ON and OFF fields are reduced by 11 and 6% in the dark reared mice. Consequently, the receptive/dendritic field ratios of ON and OFF cells change from 1.6 to 3.8 in mice raised under the cyclic light condition to 1.7 and 2.2 in the dark reared mice. For the ON and OFF fields of ON–OFF cells, the receptive/dendritic field ratios remain at 2.5 and 2.0 in mice raised under the cyclic light condition and constant darkness. Therefore, light deprivation retards the development of both dendritic and receptive fields of RGCs in a somewhat cell type-dependent manner. Interestingly, light deprivation significantly decreases the receptive/dendritic field ratios of OFF cells by reducing the number of the large-field OFF cells, suggesting that the developmental synaptic converging of large-field OFF cells depends upon visual activity.

## Data Availability Statement

The raw data supporting the conclusions of this article will be made available by the authors, without undue reservation.

## Ethics Statement

The animal study was reviewed and approved by Institutional Animal Care and Use Committee (IACUC) of the University of Utah.

## Author Contributions

HC and H-PX were in charge of the data collection, data analysis, and manuscript preparation. PW did the animal preparation and resource management. NT handled the experimental design, data analysis, manuscript preparation, and research fund management. All authors contributed to the article and approved the submitted version.

## Conflict of Interest

The authors declare that the research was conducted in the absence of any commercial or financial relationships that could be construed as a potential conflict of interest.
